# The European Diploma in Radiology (EDiR): investing in the future of the new generations of radiologists

**DOI:** 10.1007/s13244-018-0665-7

**Published:** 2018-10-05

**Authors:** European Board of Radiology (EBR)

**Affiliations:** European Board of Radiology (EBR), C/ Passeig de Gràcia, 86, 8, 08008 Barcelona, Spain

**Keywords:** Accreditation, Residency programme, Training programme, Certification, Examination

## Abstract

**Abstract:**

This review aims to describe the organisation and the content of the European Diploma in Radiology (EDiR). The EDiR examination is available to radiologists and radiology residents in their last year of training. It certifies that their levels of knowledge and competency are in line with the ESR European Training Curriculum for Radiology (ETC) of the European Society of Radiology (ESR). The EDiR is an additional qualification of excellence, which serves the standardisation and accreditation of radiologists across European borders. It provides an international benchmark for general radiology and is officially and fully endorsed by the European Union of Medical Specialists (UEMS) and the ESR. The EDiR is recognised as an equivalent of the Polish exit training examination, the first part of the Turkish board examination and the image interpretation part of the Finnish national examination. Moreover, in order to practice radiology in The Netherlands, trainees must either pass their national board examination or the EDiR. It has significant value in many other countries. The examination consists of three parts: Multiple Response Questions (MRQs), Short Cases (SCs) and the Clinically Oriented Reasoning Evaluation (CORE). The committees that form the EDiR Scientific Board follow a structured workflow to prepare each examination, ensuring an adequate peer review system for quality assurance.

**Key Points:**

• *EDiR helps to standardise radiology training.*

• *EDiR is an international certification method established across Europe.*

• *Ideally all training programmes should embrace EDiR as exit examination after completing their training period.*

• *The EDiR exam consists of multiple response questions, short cases and the Clinically Oriented Reasoning Evaluation.*

## Introduction

The European Society of Radiology (ESR) is dedicated to promoting and coordinating the scientific, educational, philanthropic, intellectual and professional activities of Radiology. Providing education and harmonising radiology training is a major goal, however not yet fully achieved. In reality, a broad range of training programmes is offered across European countries. To date, specialist certification is not standardised within Europe. While in some countries there is a national examination at the end of the residency programme, in others, a specialist certification is not mandatory or not even available [1, 2].

In light of this heterogeneous situation, the ESR created in 2011 the European Diploma in Radiology (EDiR) in order to establish a European reference standard of training. The EDiR tests for knowledge and competence in line with the contents outlined in the ESR European Training Curriculum (ETC) for Radiology [3].

The EDiR provides a certificate of standardised qualification of individual radiologists, thereby contributing to the best standards of patient care. Its target audience are certified radiologists or radiology residents in the last year of their national radiology training programme at the time of the examination. The application for the EDiR examination can be carried out individually or as a group through hospital heads of department or the corresponding national society.

## Selection of candidates

Candidates must be certified radiologists or residents in their last year of national radiology training at the time of the examination. The total length of training and supervised practical experience must be more than four years at the time of application. In cases where the length of the national radiology training programme is less than five years, experience as a supervised staff radiologist is considered.

In countries, where the national training programme consists of four years only, candidates applying via some national radiological societies or training institutions, are permitted to take the examination in their fourth year of training. This is justified by exceptional rules agreed upon between the EBR and these institutions. These agreements are authorised on a case by case basis by the EDiR Scientific Director, and are underlain thorough educational quality criteria.

In any case, EDiR diploma certificates are only awarded to successful candidates who have finished their radiology training and submitted an official certificate of training completion.

**Fig. 1:**
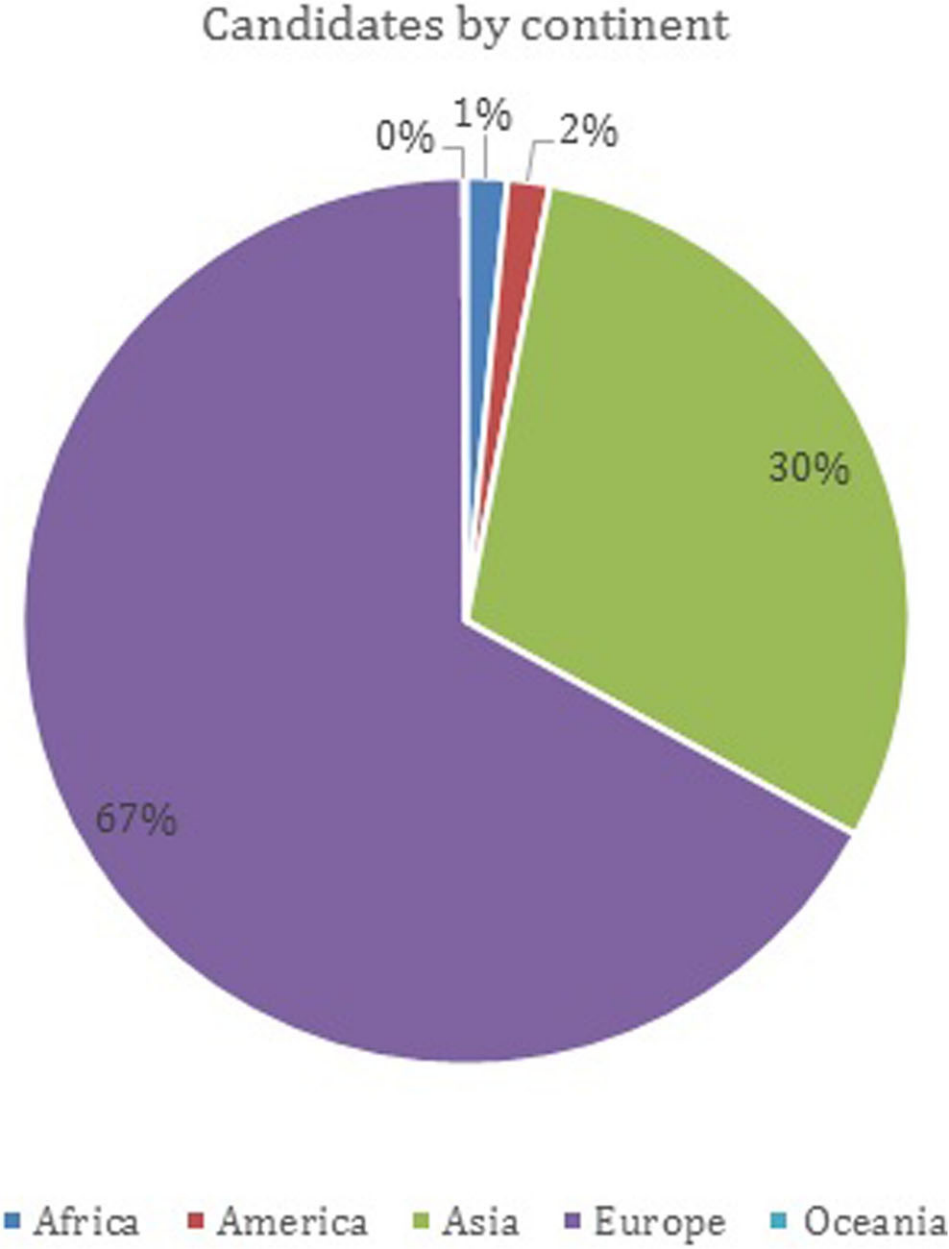
Candidates by continent

A total of 2,159 candidates have taken the examination until 2017, with a pass rate of around 70%. EDiR candidates come from all over the world: 70% are candidates from Europe and the remaining 30% from the rest of the world. In total, there are candidates from 70 different countries. Poland, Saudi Arabia, Turkey, Spain, France and Italy are the countries with the largest number of candidates (Fig. 1).

## Examination setup

EDiR examinations are conducted around the world throughout the year to suit the needs of potential EDiR candidates. The flagship examination is held during the European Congress of Radiology (ECR).

As for the content of the examination, questions are provided by the members of the Written Evaluation and CORE (Clinically Oriented Reasoning Evaluation) Committees. The Written Evaluation Committee writes, supervises and approves the first section of the examination (Multiple Response Questions and Short Cases) and the CORE Committee carries out the same tasks for the CORE cases. Both committees include subspecialists from different areas according to the EDiR blueprint, which is based on the ETC. The Written Evaluation Committee has 14 team leaders, a coordinator and a chairperson, while the CORE Committee is composed of 8 members, a coordinator and a chairperson. The coordinator is in charge of the quality of the submitted material, the editing process and its suitability for the examination. Committee members are in charge of case creation/collection.

Before the EBR Office uploads the cases onto the EDiR database, they undergo a professional proofreading. The EBR Office carries out a format-wise quality control of the submitted material to guarantee they meet the standards set by the Standards Committee (format of the questions and images, hints, abbreviations, etc.). Then, the content and the format of the cases are thoroughly revised again by the coordinator of the corresponding committee. During this second review process, special attention is paid to the following aspects:

- Ambiguous questions or answers
- Avoid relative terms such as “frequently”, “rarely” or “often”
- Do not use “always” or “never” (and no “maybe” terms such as “can sometimes” or “is often”)
- Do not use answers like “all of the above” or “none of the above”
- Do not use mutually exclusive paired options,
- The answers have to be focused (they all are diagnoses, or imaging findings, or therapies, etc.)

At this stage, the development process—which also involves a great amount of personal interaction between the EBR Office and the corresponding committees—starts. The EBR is committed to maintaining the highest standards in general radiology by adhering to strict procedures and policies. All examinations are created from scratch, reviewed and approved by the panel of experts. In order to be able to reuse a question in an examination, it must prove to have statistical value in distinguishing between excellent or inadequate expert knowledge, and that it was last used in an examination at least two years ago. Currently, the proportion of reused questions is 30–50% per examination.

The EBR Office makes a preliminary selection of the questions following the EDiR blueprint, where the relative percentage of the distribution of the content and the proportion of categories for each examination are established. The preliminary selection of cases is reviewed and approved at the cases meetings of the Written Evaluation Committee (MRQs and Short Cases) and of the CORE Committee (CORE cases). Lastly, they undergo a final quality assurance by the EDiR Scientific Director or by the respective committee chair for consistency and suitability purposes.

The EDiR Study Guide section comprises a list of educational resources as a general guidance for students. This includes a list of recommended reference textbooks and EDiR sample tests which are available on the EBR website and on the ESR website through the ESR e-learning tool Education on Demand [4].

## Examination structure

The examination is designed to test knowledge, skills and competence in anatomy, pathophysiology, imaging procedures, physics and management in general radiology [5].

The following 13 categories are included in all examinations: abdominal, breast, cardiac, chest/thorax, head and neck, imaging physics including radiation safety, imaging pharmacology including contrast media and radiopharmaceuticals, interventional and vascular, management, musculoskeletal, paediatrics, urogenital and neuroradiology (Table 1).

**Table 1:** EDiR blueprint

Subspecialty	Number of regular MRQs	Number of pictorial MRQs	Short Cases	CORE Cases
Abdominal radiology	6	3	3	1
Breast radiology	3	2	2	1
Cardiac radiology	2	1	1	1
Chest/Thorax radiology	5	3	3	1
Genitourinary radiology	5	4	4	1
Head and Neck radiology	4	2	2	1
Imaging pharmacology including contrast media and radiopharmaceuticals	2	–	–	–
Imaging physics including radiation safety	3	–	–	–
Interventional and vascular radiology	4	2	1	–
Management	2	–	–	–
Musculoskeletal radiology	6	3	3	2
Neuroradiology	5	3	3	1
Paediatric radiology	3	2	2	1
TOTAL	50	25	24	10

**Fig. 2:**
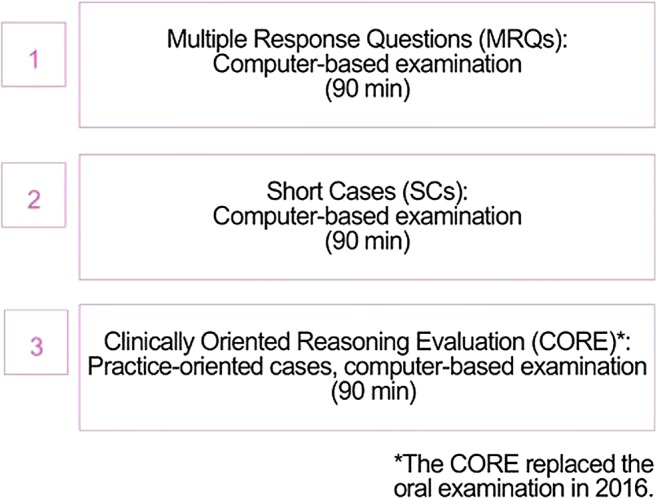
Structure of the examination

English is the main language of the examination which is divided into three parts: a knowledge test with Multiple Response Questions (MRQs) and Short Cases (SCs) and the Clinically Oriented Reasoning Evaluation (CORE) where problem-solving skills are tested (Fig. 2).

From 2011 to 2016, the examination consisted of MRQs, SCs and an oral examination that included 12 cases of different subspecialties presented by two examiners using a DICOM viewer. After 5 years of experience in 2016, the exponential growth in the number of candidates and the need to offer an online-based and even better-standardised examination system led to a move from the oral to the CORE examination. The new software, developed by the EBR office supports a fully computer-based examination, and features a DICOM viewer for case analysis, simulating the daily work of radiologists. This new examination software is not only used by the EBR, but also by some ESR subspecialty societies (e.g. the European Society of Cardiovascular Radiology (ESCR) and the European Society of Breast Imaging (EUSOBI)), as well as by international societies (e.g. the Royal Australian and New Zealand College of Radiologists (RANZCR) and the Consejo Mexicano de Radiología e Imagen (CMRI)).

A group of experts of the Universitat Autònoma of Barcelona determined that the reliability and consistency of this new examination structure is more objective and its scores more homogeneous compared to the previous oral exam.

To guarantee that third parties cannot access the questions, the EDiR software is protected by two levels of security: software security (which includes encrypted information, restricted access, and attack prevention) and server security (consisting of a firewall and protection software installed).

Questions are released to the online system not before the exam day and each candidate receives individual login data per exam to access them.

## Scoring

### SC Score

Arithmetic mean of the scores obtained in each Short Case. Each Short Case is scored from 0 to 100%.

### MRQ Score

Arithmetic mean of the scores obtained in each Multiple Response Question. Each MRQ is scored from 0 to 100%.

### CORE Score

Arithmetic mean of the scores obtained in each CORE case. Each CORE case is graded 0–10 points by the assigned examiner. Additionally, examiners can grade CORE cases with the score *unsafe*. *Unsafe* is assigned when a catastrophic error is made (in observation, interpretation or management) that would have a major impact on the patient. The *unsafe* score counts as 0 when calculating the final score.

While the first two sections of the examination (MRQs and SCs) are automatically calculated by the system, the CORE section is scored manually by the examiners who are also members of the CORE Committee. For consistency, correction criteria for all cases of an examination are reviewed and have to be unanimously approved by all examiners in a preparatory meeting taking place prior to the examination.

The pass mark is set for each particular exam and is adjusted based on its difficulty. The overall pass rate, which has proven to be stable over the years, is around 70%.

## Official certificate

The European Union of Medical Specialists (UEMS) and the ESR officially and fully endorse EDiR.

Founded in 1958, the UEMS is the oldest medical organisation in Europe and has become the representative organisation of the national associations of medical specialists in the European Union (EU) and its associated countries. The European Boards associated with the UEMS Specialists Sections are dealing with setting standards for training and education within their medical specialty. Currently, EDiR has two UEMS representatives on its committees.

To date, EDiR is recognised as the equivalent of the Polish exit training examination, the first part of the Turkish board examination and the image interpretation part of the Finnish national examination. Moreover, in order to practice radiology in The Netherlands, trainees must either pass their national board examination or the EDiR. Additionally, a Memorandum of Understanding has been signed with the Faculty of Radiologists and the Royal College of Surgeons in Ireland and with the Radiological Society of Pakistan.

Furthermore, EDiR has a significant additional value, although it is not official, in countries such as France, Italy, Belgium, Sweden, Russia, Bosnia and Herzegovina, Slovakia, Malta, Estonia, Croatia and Georgia.

However, EDiR’s commitment to excellence does not stop here. On a personal level, holding an EDiR represents an added and differential value to the radiologists’ curriculum vitae when applying for a job or a fellowship. Professional mobility is highly supported by a widely accepted European certification.

On an institutional and political level, EDiR is the ESR’s number one instrument to achieve the goal of harmonised European standards in radiology training.

**Fig. 3:**
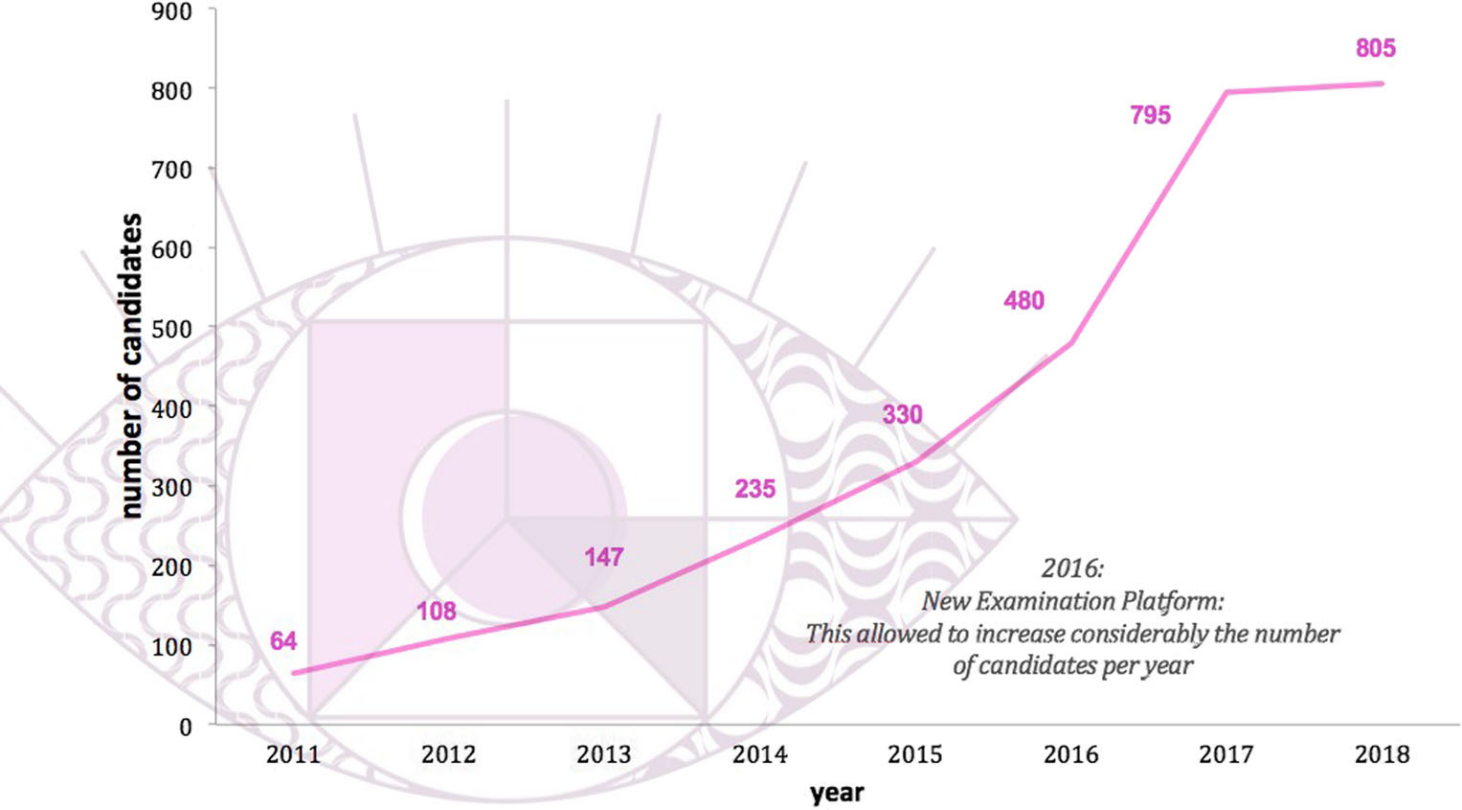
Evolution of EDiR over the years

The interest in the examination has increased exponentially since 2011. More than 2000 candidates have taken EDiR from 2011 to 2017, and this number is expected to grow substantially in the coming years due to the online-based examination structure (Fig. 3).

## Conclusion

EDiR is a statistically proven examination that covers all areas of the ETC and meets the highest quality standards.

All experts on the committees involved are established radiologists, and are assisted by the custom software and scoring system. Together with the EBR Office, they strive to further improve the quality and the consistency of the examination day by day. EDiR stands for a benchmark of excellence in radiology training and certifies an outstanding level of competence to the EDiR Holders.
